# An Optimized Preparation Method for Long ssDNA Donors to Facilitate Quick Knock-In Mouse Generation

**DOI:** 10.3390/cells10051076

**Published:** 2021-04-30

**Authors:** Yukiko U. Inoue, Yuki Morimoto, Mayumi Yamada, Ryosuke Kaneko, Kazumi Shimaoka, Shinji Oki, Mayuko Hotta, Junko Asami, Eriko Koike, Kei Hori, Mikio Hoshino, Itaru Imayoshi, Takayoshi Inoue

**Affiliations:** 1 Department of Biochemistry and Cellular Biology, National Institute of Neuroscience, National Center of Neurology and Psychiatry, Ogawahigashi, Kodaira, Tokyo 187-8502, Japan; 99a117@gmail.com (Y.M.); kshimaoka@ncnp.go.jp (K.S.); mhotta@ncnp.go.jp (M.H.); asami@ncnp.go.jp (J.A.); koikeike@ncnp.go.jp (E.K.); khori@ncnp.go.jp (K.H.); hoshino@ncnp.go.jp (M.H.); 2 Research Center for Dynamic Living Systems, Graduate School of Biostudies, Kyoto University, Kyoto 606-8501, Japan; yamada.mayumi.4a@kyoto-u.ac.jp (M.Y.); imayoshi.itaru.2n@kyoto-u.ac.jp (I.I.); 3 KOKORO-Biology Group, Laboratories for Integrated Biology, Graduate School of Frontier Biosciences, Osaka University, Suita, Osaka 565-0871, Japan; rkaneko@fbs.osaka-u.ac.jp; 4 Department of Immunology, National Institute of Neuroscience, National Center of Neurology and Psychiatry, Ogawahigashi, Kodaira, Tokyo 187-8502, Japan; soki@ncnp.go.jp; 5 Department of Deconstruction of Stem Cells, Institute for Frontier Life and Medical Sciences, Kyoto University, Kyoto 606-8507, Japan

**Keywords:** long ssDNA, phospho-PCR, knock-in, CRISPR/Cas9

## Abstract

Fluorescent reporter mouse lines and Cre/Flp recombinase driver lines play essential roles in investigating various molecular functions *in vivo*. Now that applications of the CRISPR/Cas9 genome-editing system to mouse fertilized eggs have drastically accelerated these knock-in mouse generations, the next need is to establish easier, quicker, and cheaper methods for knock-in donor preparation. Here, we reverify and optimize the phospho-PCR method to obtain highly pure long single-stranded DNAs (ssDNAs) suitable for knock-in mouse generation via genome editing. The sophisticated sequential use of two exonucleases, in which double-stranded DNAs (dsDNAs) amplified by a pair of 5′-phosphorylated primer and normal primer are digested by Lambda exonuclease to yield ssDNA and the following Exonuclease III treatment degrades the remaining dsDNAs, enables much easier long ssDNA productions without laborious gel extraction steps. By microinjecting these donor DNAs along with CRISPR/Cas9 components into mouse zygotes, we have effectively generated fluorescent reporter lines and recombinase drivers. To further broaden the applicability, we have prepared long ssDNA donors in higher concentrations and electroporated them into mouse eggs to successfully obtain knock-in embryos. This classical yet improved method, which is regaining attention on the progress of CRISPR/Cas9 development, shall be the first choice for long donor DNA preparation, and the resulting knock-in lines could accelerate life science research.

## 1. Introduction

Fluorescent reporter mouse lines and site-specific recombinase driver lines play pivotal roles to elucidate molecular functions in vivo. Fluorescent proteins fused to or bicistronically expressed with gene products of biological interest can visualize/recapitulate the endogenous expression profiles, help manipulate the cells on site (for example, patch-clamp recordings), and assist fluorescence-activated cell sorting (FACS). Similarly, Cre/Flp recombinase expressing lines are employed for conditional knock-out mouse generations and extensively support various latest techniques such as optogenetics or chemogenetics in combination with viral vectors.

The recent development of the clustered regularly interspaced short palindrome repeats (CRISPR)/CRISPR-associated protein-9 nuclease (Cas9) genome editing system has totally changed the knock-in mouse generation procedures that had relied on the gene targeting in embryonic stem cells [1]. To exploit this system for reporter/recombinase knock-in mouse generations, we only need to directly deliver CRISPR components (guide RNAs and Cas9 nucleases) along with the donor DNAs into mouse zygotes by microinjection or electroporation [2,3]. While chemically synthesized dual guide RNAs, CRISPR RNA (crRNA) and trans-activating crRNA (tracrRNA), can be easily ordered in ready-to-use form and recombinant Cas9 nuclease proteins are available from manufacturers, researchers still have needed to spend much more time and cost in preparing knock-in donor DNAs, especially when they desire to create a long gene cassette knock-in allele.

Conceptually, the CRISPR/Cas9 system introduces a targeted DNA double-strand break (DSB) into the genome, and the genome editing strategy harnesses the endogenous DNA repair processes within the cells to yield desired genomic alterations [4,5]. In order to integrate fluorescent reporter or Cre/Flp recombinase sequences into the target locus, researchers employ homology-directed repair (HDR). HDR is broadly classified into two pathways by the nature of nucleic acid template: homologous recombination (HR) using dsDNA donors and single-stranded template repair (SSTR) utilizing ssDNA donors [4,5]. The SSTR-based knock-in strategy has become popular because of its high efficiency [6]. The detailed mechanisms for this pathway have been analyzed in the recent article [7]. Being inspired by the fact that chemically synthesized ssDNAs can effectively work as repair templates for single base substitution or epitope tagging, researchers have also successfully exploited long ssDNAs for the longer gene cassette knock-in [8,9,10,11].

Whereas ssDNAs up to 200 nucleotides can be easily purchased at low cost, researchers still need to manually prepare long ssDNA donors in the lab, and two already reported methods for long ssDNA preparation are somewhat complicated. The first is referred to as the ivTRT method (Supplementary Figure S1A) [8,10,12] in which the dsDNA template is transcribed into RNA, then reverse transcription is performed to yield RNA/DNA hybrids. By degrading the RNA strand by RNase treatment, long ssDNA donors can be prepared. The other is called the nicking endonuclease method (Supplementary Figure S1B) [9,11], in which the dsDNA plasmid is double-nicked and denatured, then subjected to electrophoresis to separate long ssDNA fragments. Although the long ssDNA donors produced by these two methods could be successfully used for knock-in mouse generations, the last purification procedure where the ssDNAs were required to be cut out and extracted from agarose gels have remained laborious.

**Figure 1:**
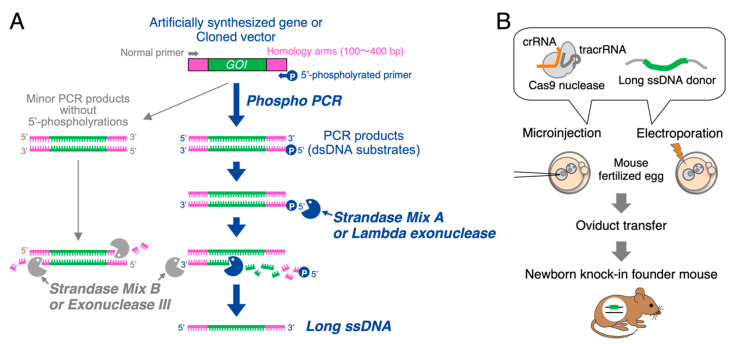
Schematic diagram of knock-in mouse generation using long ssDNA donors prepared by the phospho-PCR method. (**A**) Flowchart of the phospho-PCR method. A dsDNA template containing a gene cassette of interest (GOI) flanked by homology arms on both sides is depicted at the top. A pair of normal primer and 5′-phospholyrated primer for PCR is used to amplify the starting dsDNA substrates for exonuclease reactions. Strandase Mix A or Lambda exonuclease selectively degrades the phosphorylated chains of the duplex. Strandase Mix B or Exonuclease III subsequently help complete the selective degradations to yield long ssDNAs. Minor PCR products without 5′-phosphorylations are also extensively digested by Exonuclease III activities, whose efficiency is detailed in Results Section 3.2. Note that this sequential use of two exonucleases enables highly pure ssDNA production. (**B**) Schematic of CRISPR/Cas9 mediated knock-in mouse generation using long ssDNA donors. Long ssDNA donor and Cas9-guide RNA complex can be introduced into mouse fertilized eggs either by pronuclear injection or by electroporation. Treated eggs are transferred into the oviduct of pseudo-pregnant recipients to obtain knock-in founders.

One more strategy called the phospho-PCR method (Supplementary Figure S1C) has been proposed and domestically prevailed in Japan [13,14,15], but the detailed conditions for knock-in mouse generations have not been fully evaluated. Classically, ssDNA molecules play a key role in various techniques such as DNA sequencings [16,17,18], single nucleotide polymorphism (SNP) analyses [19], and DNA aptamer selections [20]. For these applications, selective strand digestions of dsDNA utilizing specific exonucleases have been employed to prepare ssDNAs [21,22]. Lambda exonuclease is a highly processive 5′ to 3’ dsDNA exonuclease that selectively degrades a phosphorylated chain of the duplex to yield ssDNA [17] (Figure 1A). Exonuclease III, on the other hand, catalyzes the stepwise removal of mononucleotides from 3’-hydroxyl termini of duplex DNA [16] (Figure 1A). For the long ssDNA production, dsDNA substrates amplified by a pair of 5′-phosphorylated primer and normal primer are digested by Lambda exonuclease to yield ssDNA and the following Exonuclease III treatment degrades the remaining dsDNAs [14].

Here, we select the mouse *Dcx (doublecortin)* locus as a model site for Cre recombinase sequence insertion to optimize the phospho-PCR method. We accordingly start with a commercially available kit to reverify the conditions for long ssDNA productions suitable for knock-in mouse generations, then clarify the reason why this method allows us to obtain highly pure ssDNAs without laborious gel separation/extraction steps. Next, by microinjecting the long ssDNA donors prepared by this method along with CRISPR/Cas9 components into mouse zygotes, we show that these donors effectively work as repair templates for reporter/recombinase knock-in mouse generations. Furthermore, we present that these long ssDNA donors can be utilized for electroporation-mediated knock-in mouse generations, making the procedures much easier for researchers by eliminating microinjection steps. Collectively, we show that this classical, yet improved method shall be the first choice for long donor DNA preparation, and the resulting knock-in lines could accelerate life science research. 

## 2. Materials and Methods

### 2.1. Animals

All animal care and experimental procedure were performed in accordance with the Guidelines for Animal Experiments approved by the Ethics Committee in the National Institute of Neuroscience (approved number: 2017005 (approved on 3 March 2017), 2020007 (approved on 26 February 2020)). B6C3F1 mice for fertilized eggs collection and ICR mice as surrogate mothers were purchased from SLC Japan.

### 2.2. Preparation of CRISPR Components

In order to insert reporter/recombinase sequences into the loci of interest (listed in the tables of Results Section 3.3) via the CRISPR/Cas9 system, we analyzed the targeted regions by using the web-based CRISPR design tool, CRISPOR [23] (http://crispor.tefor.net/) (accessed on 29 April 2021), to select the guide RNA sequences closest to the insertion sites with off-target effects as minimum as possible. Two parts of the CRISPR guide RNA, crRNA and tracrRNA (depicted in Figure 1B and listed in Table S1), were chemically synthesized (FASMAC or Integrated DNA Technologies). For pronuclear injections, equimolar crRNA and tracrRNA were mixed and annealed (94 °C, 2 min then at room temperature for 10 min) to generate crRNA/tracrRNA complex. Recombinant Cas9 protein (EnGen Cas9 NLS S.pyogenes from NEB or Alt-R S.p. HiFi Cas9 Nuclease V3 from Integrated DNA Technologies) was purchased from the suppliers.

### 2.3. Preparation of Long ssDNA Donors by the Phospho-PCR Method

Long ssDNA donors were produced by using a commercially available kit, Guide-it Long ssDNA Production System (Takara Bio, Kusatsu, Shiga, Japan, 632644, 632666) (Figure 1A), basically following the manufacturer’s instruction [24] yet with some modifications (the modified points are described in Results Section 3.1). First, dsDNA templates containing a gene cassette of interest (GOI) flanked by 102–413 base pair (bp) homology arms on both sides were prepared by artificial gene syntheses (eurofins Genomics) or the In-Fusion Cloning method (Takara Bio). Tips for dsDNA template designs are described in Supplementary Information 1. Next, the starting dsDNA substrates for exonuclease reactions were amplified with a pair of 5′-phosphorylated primer and normal primer by using the high-fidelity PCR enzyme (PrimeSTAR Max DNA polymerase, Takara Bio). The PCR products were purified by NucleoSpin column (Takara Bio). Then, the phosphorylated chains of the duplex were selectively degraded by Strandase Mix A (37 °C, 5 min for 1000 bp long, followed by heat deactivation at 80 °C, 5 min) to obtain ssDNAs. Next, to complete the selective degradation, we added Strandase Mix B to the reaction mixture (37 °C, 5 min for 1000 bp long, followed by heat deactivation at 80 °C, 5 min). A small portion of the reaction mixture was checked on the agarose gel electrophoresis to confirm the ssDNA purity, and then the remaining mixture was purified by NucleoSpin column (Takara Bio) to obtain ssDNAs. The amounts of input dsDNA (µg) and output ssDNA (µg) are listed in the table of Results Section 3.2. To obtain a large amount of ssDNA as a concentrated solution suitable for electroporation, we scaled the initial PCR mixtures up to 5–6 times’ volume, and the exonuclease reactions were performed using several tubes in parallel. The resulting ssDNAs were gathered into one tube and concentrated by isopropanol precipitation.

To manually produce long ssDNA donors without using the commercial kit, we treated dsDNA substrates prepared through the same PCR procedures described above with Lambda exonuclease (New England BioLab, Ipswich, MA, USA, M0262, 10 units for 10 µg dsDNA) at 37 °C for 30 min to degrade 5′-phospholyrated chains of dsDNAs followed by heat deactivation at 75 °C, 10 min. Then, Exonuclease III (Takara Bio 2170A, 90 units for 10 µg dsDNA) was added to the mixture (37 °C, 5 min for 1500 bp, followed by heat deactivation at 65 °C, 5 min) to complete the ssDNA production. The resulting ssDNA was purified by NucleoSpin column (Takara Bio) after confirming the purity by electrophoresis. Detailed protocols for this manual ssDNA production are described in Supplementary Information 3.

### 2.4. Optimization of PCR Conditions to Amplify Pure Single Products as Exonuclease Substrates

To avoid unwanted non-specific amplifications, we precisely optimized the primers’ concentrations, the annealing temperature, and the templates’ concentrations for high-fidelity PCR enzyme, PrimeSTAR Max DNA polymerase (described in Results Section 3.1). To this end, the primers’ concentrations were reduced to one-fourth (0.8 µM to 0.2 µM), and the annealing temperature was raised from 55 °C to 58 °C. The templates’ concentrations were rigorously reduced in a stepwise manner (0.25 ng/µL, 0.25 pg/µL, 0.25 fg/µL, and 0.025 fg/µL) to find the best point for clear single product. Further tips for PCR optimization are described in Supplementary Information 2.

### 2.5. Comparison of Exonuclease Activities between the Commercially Available Kit and the Manual Protocol

To compare the exonuclease activity contained in Strandase Mix A from the commercial kit (Takara Bio) with the activity of Lambda exonuclease (NEB), we digested 2 µg of dsDNA substrates amplified by a pair of 5′-phospholyrated primer and normal primer for the *Dcx* locus solely with either Strandase Mix A or Lambda exonuclease (described in Results Section 3.2). Strandase Mix A was added to the dsDNA in accordance with the manufacturer’s protocol described in Section 2.3. Lambda exonuclease was added to the dsDNA (2 units for 2 µg of dsDNA), and the mixture was incubated at the conditions described in Section 2.3. Similarly, to compare the exonuclease activity contained in Strandase Mix B with the activity of Exonuclease III (Takara Bio), we sequentially treated 2 µg of dsDNA substrates amplified above with either Strandase Mix A followed by Strandase Mix B or with Lambda exonuclease followed by Exonuclease III (described in Results Section 3.2). Strandase Mix A and B were added to the dsDNA by following the manufacturer’s instructions. Exonuclease III was sequentially added to the Lambda exonuclease reaction mixture (18 units for 2 µg of the starting dsDNA), and the mixture was incubated at the conditions described in Section 2.3. These four types of reaction mixtures were analyzed by the agarose gel electrophoresis to visualize the resulting long ssDNAs and the remaining dsDNA substrates.

### 2.6. Microinjection of Long ssDNA Donors into Mouse Fertilized Eggs

Cas9 protein, crRNA/tracrRNA complex, and long ssDNA donor were mixed in 0.1× TE buffer (10 mM Tris-HCl buffer (pH 7.6), 0.1 mM EDTA (pH 8.0)) at the working concentrations of 50–100 ng/µL, 35–50 ng/µL, and 17–27 ng/µL, respectively. One-cell-stage zygotes were obtained by mating B6C3F1 stud males with super-ovulated females. Pronuclear injections and mouse transgenesis experiments were performed by standard protocols [25]. The detailed microinjection conditions and results are listed in the tables of Results Section 3.3.

### 2.7. PCR Genotyping and Sequencing Analyses

Genomic DNAs were prepared from newborns’ tail by proteinase K treatment in Lysis Buffer (Takara Bio). Knock-in mice were screened by PCR using Tks Gflex DNA polymerase (Takara Bio) or PrimeSTAR GXL DNA polymerase (Takara Bio). Two pairs of primers for each locus were designed external to the donor DNA’s homology arms so as to exclude the detection of unintended random integrations. Primers used for Dcx-T2A-iCre knock-in (described in Results Section 3.3) and Pax6-mCitrine knock-in (described in Results Section 3.4) are listed in Table S2. For knock-in candidates, the PCR products were analyzed by Sanger sequencing to confirm carrying the correct insertions. Only sequence-verified knock-in founders were counted as knock-in pups (KI) in the table of Results Section 3.3.

### 2.8. Electroporation of Long ssDNA Donors into Mouse One-Cell Embryos

Cas9 protein, crRNA, tracrRNA, and long ssDNA donor were mixed in Opti-MEM I (Thermo Fisher, Waltham, MA, USA, 31985) at the working concentrations of 100 ng/µL, 200 ng/µL, 200 ng/µL, and 350–1000 ng/µL, respectively. The concentrations of Cas9 protein, guide RNAs, and ssDNA donors were 2 times higher than in the previous report [3]. Genome Editor (BEX, Tokyo, Japan, GEB15) and the plate-type electrode (BEX, LF501PT1-10) were used for the electroporation. Three repeats of square pulse, Pd V (voltage of the pulses): 50 V, Pd A (current of the pulses): 290–320 mA, Pd on: 5 msec and Pd off: 50 msec, were applied to fertilized eggs. These electrical conditions were largely modified to be suitable for long ssDNA donors from the previously reported protocols generally used for short ssDNA donors [3].

For Pax6-mCitrine knock-in (described in Results Section 3.4), electroporated eggs were transferred into the oviducts of pseudo-pregnant recipients to obtain embryonic day 10.5 (E10.5) embryos. The mCitrine fluorescence was detected by using BZ-X800 (KEYENCE, Osaka, Japan). Yolk sacs were sampled into microtubes, treated with proteinase K in Lysis Buffer, and used as PCR templates for genotyping.

For Dcx-T2A-iCre knock-in (Supplementary Figure S2), electroporated eggs were cultured in KSOM medium for 3 days to obtain blastocysts. Blastocysts were separately sampled into microtubes and lysed by adding 0.01% SDS and 0.007 N NaOH. The mixtures were neutralized with 4 mM Tris-HCl buffer (pH 8.3) and used as PCR templates for genotyping.

## 3. Results

### 3.1. Optimization of PCR Conditions to Obtain a Clear Single Product, Which Was the Critical First Step for Pure ssDNA Production

**Figure 2:**
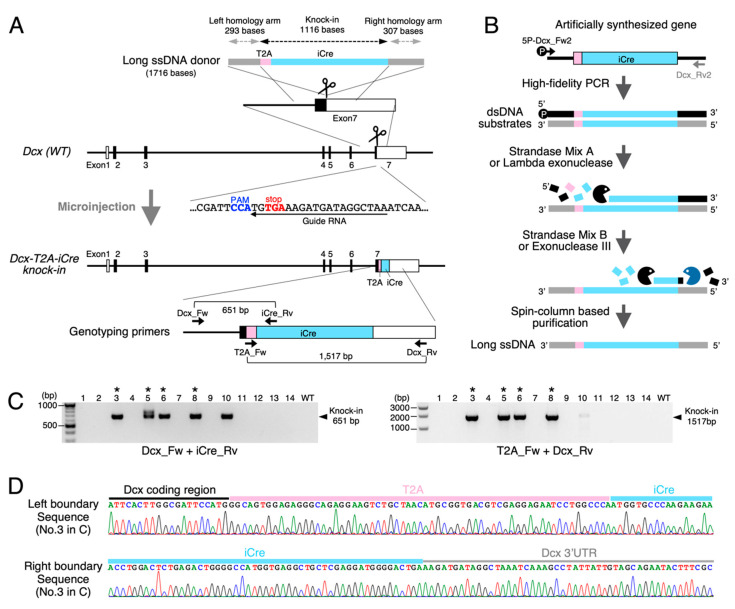
Mouse Dcx locus as a model site for targeted recombinase knock-in. (**A**) Targeting strategy to insert an iCre recombinase cassette just upstream from the translational stop codon in the Dcx gene is outlined. Schematic of the Dcx gene structure, the guide RNA sequences, and the long ssDNA donor is shown in the upper half. The resulting knock-in allele and its genotyping primers are depicted in the lower half. T2A peptide sequences were employed for bicistronic iCre expressions in Dcx-expressing cells. (**B**) The flowchart of long ssDNA production for *Dcx-T2A-iCre* knock-in via the phospho-PCR method is depicted. The artificially synthesized gene containing a T2A-iCre cassette was used as the template for phospho-PCR. 5′-phosphorylated strands of the PCR products were sequentially digested by Lambda exonuclease and Exonuclease III to yield long ssDNA donor. (**C**) PCR screening results for knock-in newborns derived from the pronuclear injection shown in Table 2 (Dcx-T2A-iCre) are summarized. Two pairs of primers depicted in (**A**) were used to confirm the designed knock-in. Dcx_Fw and Dcx_Rv were designed outside from the donor DNA’s homology arms to exclude the detection of unintended random integrations. Asterisks indicate the knock-in founders with correct insertions (No.3, No.5, No.6, and No.8). No.5 carried an additional incorrect knock-in allele. (**D**) Boundary sequences between the *Dcx* gene and T2A-iCre cassette analyzed by using the genome DNA from No.3 founder in (**C**) are aligned.

In the present study, we chose the mouse *Dcx* locus on chromosome X as a main model site for Cre recombinase sequence insertion to optimize the phospho-PCR-mediated long ssDNA donor production (Figure 2A). As Dcx was reported to express in immature newborn neurons [26,27], its recombinase driver mouse line could be utilized to manipulate variety of exogenous gene expressions by combining with Cre-dependent viral vectors to examine the effects on neural circuit development and higher-order brain functions.

To bicistronically express the codon-improved version of Cre recombinase (iCre) in Dcx-expressing cells, we employed T2A peptide sequences and designed the T2A-iCre cassette insertion just upstream from the translational stop codon TGA (Figure 2A). We selected the guide RNA sequences closest to the termination codon in Exon 7. For the long ssDNA donor preparation by the phospho-PCR method, we designed 5′-phosphorylated primer (5P-Dcx-Fw2) and normal primer (Dcx_Rv2) (Figure 2B).

**Figure 3:**
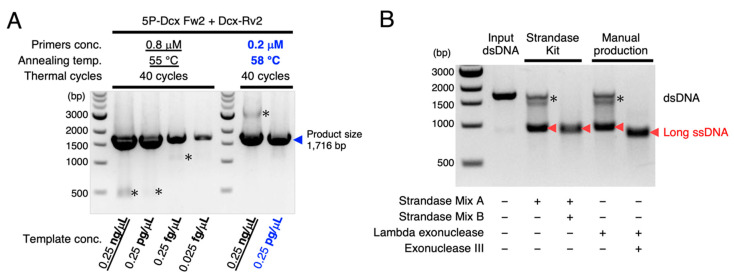
Purely amplified PCR products and the following sequential use of two exonucleases enabled high-quality ssDNA production. (**A**) Optimization of PCR conditions to obtain a clear single product was the first key step for phospho-PCR-mediated ssDNA production. Primers’ concentrations, annealing temperature, and templates’ concentration for the PCR reaction depicted in Figure 2B were optimized. The manufacturer’s protocols are underlined, and our optimized conditions are colored in blue. To obtain a single product with high efficiency, the primers’ concentrations should be lower, the annealing temperature should be higher, and the templates’ concentration should be much lower than the manufacturer’s instructions. Asterisks (*) indicate the unwanted non-specific amplifications. Blue triangle indicates the purely amplified products. (**B**) Sequential use of two exonucleases depicted in Figure 2B allowed for highly pure long ssDNA production. While the single use of Strandase Mix A or Lambda exonuclease selectively degraded 5′-phosphorylated strands to yield ssDNAs (red triangles), dsDNA substrates indicated by asterisks remained in the reaction mixtures. Subsequently added Strandase Mix B or Exonuclease III degraded the remaining dsDNAs to yield pure long ssDNAs. Note that the long ssDNA treated with Exonuclease III was slightly shorter than that treated with Strandase Mix B.

In order to generate long ssDNA donor, we first made use of a commercially available kit (Guide-it Long ssDNA Production System, Takara Bio). The initial procedure was to purely amplify dsDNA substrates for exonuclease reactions by using the high-fidelity PCR enzyme, which was the critical step for high-quality ssDNA production. However, with regard to the *Dcx-T2A-iCre* knock-in donor, we noticed that the manufacturer’s instruction did not fit this requirement (Figure 3A). At the primer concentration 0.8 µM and the annealing temperature 55 °C, the instructed template concentration 0.25 ng/µL was too high to obtain a single product. Reducing the template concentration to 0.25 pg/µL and 0.25 fg/µL did not work to suppress the non-specific amplifications. We finally obtained a single product at the template concentration 0.025 fg/µL, but the total dsDNA yield was not enough for exonuclease reactions (3.6 µg from 100 µL PCR mixture). Next, we reduced the primer concentration to 0.2 µM and raised the annealing temperature to 58 °C. This trial was successful in terms of avoiding the non-specific amplification. At the template concentration 0.25 pg/µL, we could obtain a clear single product with enough yield (11.5 µg from 100 µL PCR mixture). For the loci other than *Dcx*, we found that similar PCR conditions could be applicable. We thus finalized the primer concentration to be one-fourth lower, the annealing temperature to be higher, and the template concentration to be much lower than the manufacturer’s protocols in obtaining clear single products with enough yield. As this optimization step is essential for the successful long ssDNA production by the phospho-PCR method, we provide some more details for this step in Supplementary Information 2.

### 3.2. Sequential Exonuclease Reactions Allowed For Highly Pure Long ssDNA Production without Electrophoretic Separation Steps

By sequentially treating the dsDNA substrates amplified above with Strandase Mix A and Strandase Mix B from the commercial kit, we were able to successfully obtain the pure long ssDNA (Figure 3B) that could be easily purified using spin-column. We then sought to clarify the reason why this method could obviate the electrophoretic separation step to purify ssDNA.

Based on the generally known substrate specificities of exonucleases, we speculated that the Strandase Mix A could contain Lambda exonuclease activity and Strandase Mix B could be comprised of Exonuclease III activity. To verify this prediction, we amplified dsDNA substrates by using 5P-Dcx_Fw2 and Dcx_Rv2 primers (Figure 2B), then treated the substrates with enzymes in four combinations, as shown in Figure 3B. Although we were able to obtain long ssDNAs by treating the substrates solely with Strandase Mix A or Lambda exonuclease, there remained dsDNAs needed to be pruned (Figure 3B). On the other hand, when we sequentially added Strandase Mix B or Exonuclease III after the digestions of Strandase Mix A or Lambda exonuclease, highly pure ssDNAs were generated without any undigested leavings (Figure 3B).

As Lambda exonuclease selectively digests 5′-phosphorylated strand of dsDNA and has low activity on non-phosphorylated strand or ssDNA (Figure 1A), it has been classically employed to prepare ssDNAs for various biological techniques [17,21,22]. On the other hand, because Exonuclease III catalyzes the stepwise removal of mononucleotides from 3’-hydroxyl termini of dsDNA and does not act on ssDNA or 3’-protoruding termini, the objective ssDNAs formerly generated by Lambda exonuclease are resistant to this enzyme (Figure 1A). Exonuclease III not only helps digesting the 5′-phosphorylated strands from its 3’-termini, but also degrades the minor PCR products without 5′-phosphorylation (Figure 1A). This elegant sequential use of two enzymes would hence make it possible to produce long ssDNA donors easily and quickly.

One of the notable advantages of the phospho-PCR method was the relatively high ssDNA recovery rate (approximately 20%), as summarized in Table 1. While the nicking endonuclease method needed 100 µg plasmid DNA to obtain 2 µg ssDNA (Supplementary Figure S1B) [11], the phospho-PCR method only needed 10 µg dsDNA to yield 2 µg ssDNA (Supplementary Figure S1C and Table 1). Furthermore, we confirmed that 2699 base long ssDNA donor could be stably produced by this method (*Oxtr-T2A-iCre-ERTT2*, in Table 1), indicating that the upper length limit in this strategy could be longer than that in the ivTRT (approximately 1500 bases) [12].

### 3.3. Efficient and Quick Knock-In Mouse Generation Could Be Achieved by Using Long ssDNA Donors Prepared with the Phospho-PCR Method

By microinjecting the long ssDNA donor prepared above along with the CRISPR components into mouse zygotes (Figure 1B), we confirmed that *Dcx-T2A-iCre* knock-in founders were effectively generated (Figure 2C,D and Table 2). We used two sets of primers depicted in Figure 2A for the PCR screening and found that 4 pups out of 14 could carry the correct knock-in allele. We further analyzed the PCR products by Sanger sequencing to confirm that four pups carried the accurate T2A-iCre insertion (1116 bp) at the designed site. The efficiency of targeted insertion (28.5%) fell within the expected range previously reported in the ivTRT-mediated *Easi*-CRISPR method [10].

As summarized in Table 1 and Table 2, we applied this approach to other loci (*Tubb3*, *Tbr2*, and *Oxtr*) to insert T2A-iCre sequences, and successfully generated knock-in founders. We also integrated the codon-optimized version of enhanced flippase (FLPo) sequences along with T2A peptide sequences (T2A-FLPo; 1326 bp) into two loci (*Dcx* and *Tubb3* in Table 1 and Table 2). Furthermore, we showed that the targeted insertion of ligand-dependent chimeric Cre recombinase sequences could be achieved by employing the phospho-PCR-mediated long ssDNA donor. The gene cassette comprised of T2A peptide and iCre fused to a mutated human estrogen receptor ligand-binding domain (T2A-iCre-ERT2; 2160 bp) could be integrated into the *Oxtr* locus by this strategy (Table 1 and Table 2), whose length was longer than the maximum that could be dealt with the *Easi*-CRISPR method [10,12].

Next, we applied phospho-PCR-mediated long ssDNA donors to generate fluorescent reporter knock-in mice. As summarized in Table 1 and Table 2, by microinjecting long ssDNA donors along with CRISPR components into mouse fertilized eggs, we were able to successfully insert various fluorescent protein sequences (*EGFP*, *d2EGFP*, *DsRed*, and *tdTomato*) into four loci (*Nr4a2*, *Tbr2*, *Cdh11* [28], and *Pcdhb19*). The longest knock-in length was 1497 bp for T2A-tdTomato, which could cover any fluorescent reporter sequences. For *Pcdhb19-T2A-EGFP* knock-in, we utilized the long ssDNA donor manually produced by the combination of Lambda exonuclease and Exonuclease III, again confirming the integrity of ssDNA prepared without a commercial kit.

While the efficiencies of recombinase/fluorescent reporter knock-in varied from 5.9% to 40.0%, probably due to the locus-dependent and/or guide RNA’s activity-dependent factors, they were still enough for practical use since we could always obtain at least one founder from one trial using 36–100 fertilized eggs (Table 2).

### 3.4. Concentrated Long ssDNA Donors Suitable for the Electroporation-Mediated Genome Editing Could Be Easily Produced by the Phospho-PCR Method

**Figure 4:**
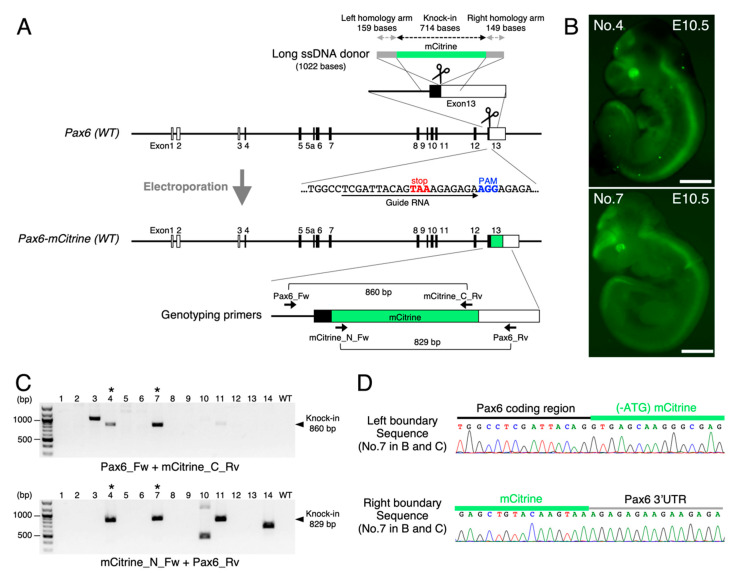
Long ssDNA donors prepared by the phospho-PCR method could also be applied to electroporation-mediated knock-in mouse generations. (**A**) Targeting strategy to integrate fluorescent reporter mCitrine sequences just upstream from the translational stop codon in the *Pax6* gene is outlined. Schematic of the *Pax6* gene structure, the guide RNA sequences, and the long ssDNA donor is shown in the upper half. The resulting knock-in allele and its genotyping primers are depicted in the lower half. (**B**) mCitrine expressions in the E10.5 knock-in embryos recapitulated the endogenous Pax6 expression patterns. No.4 and No.7 embryos in C are arranged. mCitrine-positive cells resided in the eyes, telencephalon, diencephalon, and spinal cord, where the endogenous Pax6 was expressed. Scale bar, 1 mm. (**C**) PCR screening results for knock-in embryos derived from the electroporation. Two pairs of primers depicted in A were used to confirm the designed knock-in. Pax_Fw and Pax6_Rv were designed outside from the donor DNA’s homology arms to exclude the detection of unintended random integrations. Asterisks indicate the knock-in embryos with correct insertions (No.4 and No.7). Note that some embryos other than No.4 and No.7 yielded the shorter or longer PCR products compared with the accurate size, indicating the incomplete editing. (**D**) Boundary sequences between the *Pax6* gene and mCitrine cassette analyzed by using the genome DNA extracted from No.7 embryo’s yolk sac are aligned.

To further extend the range of application, we prepared long ssDNA donors in higher concentrations suitable for electroporation-mediated genome editing to easily obtain recombinase/fluorescent reporter knock-in lines. First, we selected the *Pax6* locus to insert a fluorescent reporter, mCitrine sequences (Figure 4A). As Pax6 is strongly expressed in the eye and brain at the early developmental stage to play pivotal roles in eye development and brain patterning [29], we speculated that mCitrine knock-in could be immediately distinguishable by looking at the embryos without genotyping. To fuse mCitrine protein to the C-terminus of Pax6, we selected guide RNA sequences closest to the termination codon TAA in the Exon 13 (Figure 4A). Referring to *Easi*-CRISPR in which 55–105 base homology arms were recommended [12], we designed the long ssDNA donor with shorter arms (159 and 149 bases, Figure 4A) than those in our previous experiments (Table 2). To obtain the concentrated long ssDNA for electroporation, we simply scaled up the total volume of starting PCR mixture. Tips for this procedure are described in Supplementary Information 2. By electroporating the long ssDNA donor along with the CRISPR components into mouse zygotes (Figure 1B), we successfully observed the fluorescent reporter expressions in two embryos at E10.5 (Figure 4B, No.4 and No.7), which recapitulated the endogenous Pax6 expression profiles. We used two sets of primers depicted in Figure 4A for the PCR genotyping and found that No.4 and No.7 could carry the correct knock-in allele (Figure 4C). We further analyzed the PCR products by Sanger sequencing to confirm that two embryos carried the accurate mCitrine insertion (714 bp) at the targeted site (Figure 4D).

Next, to determine the maximum length for electroporation using the phospho-PCR-mediated long ssDNA donor, we tried T2A-iCre cassette insertion into the *Dcx* locus (Supplementary Figure S2A), which was feasible by microinjection in Figure 2. After the electroporation of long ssDNA donor along with the CRISPR components, we cultured the mouse eggs for 3 days to obtain blastocysts. We separately sampled the blastocysts into microtubes and used them as the PCR templates for genotyping. As for the results, we found that 4 blastocysts out of 40 could harbor the correct knock-in allele (Supplementary Figure S2B). We further analyzed the PCR products by Sanger sequencing to confirm that four blastocysts carried the accurate T2A-iCre insertion (1116 bp) at the targeted site (Supplementary Figure S2C), indicating that a gene cassette more than 1100 bp long could be integrated by our new strategy.

## 4. Discussion

Development of the CRISPR/Cas9 system has allowed us to directly manipulate the genome in mouse zygotes with extremely high efficiencies, realizing the ultra-rapid one-step generations of genetically modified mouse lines without embryonic stem cells. Upon this remarkable paradigm shift in mouse transgenesis, preparation methods for long donor DNAs have still been needed to be refined.

In this study, we optimized the phospho-PCR method to obtain highly pure long ssDNA donors suitable for knock-in mouse generations. Although the usefulness of long ssDNA donors prepared by this method in cultured cell experiments had been previously reported [30,31], our *in vivo* study demonstrated the applicability of this strategy to mouse zygotes for the first time with sufficient numbers of practical examples.

The main advantage of the phospho-PCR method is in its simple and speedy procedures with no RNA works or DNA denaturing steps. While the previously reported long ssDNA production methods such as the ivTRT [8,10,12] or the nicking endonuclease method [9,11] require electrophoretic separations and gel extractions to purify the ssDNA accompanied with a large amount of DNA loss, the phospho-PCR method only needs spin-columns for the purification. This merit is particularly important when we manage to prepare concentrated long ssDNA donors for electroporation. In addition, the maximum length for long ssDNA production in this strategy could be longer than that in the ivTRT (approximately 1500 bases) because of the highest fidelity of the PCR enzyme compared with the strictness of two enzymes used in the ivTRT, namely, T7 RNA polymerase and reverse transcriptase [8]. In fact, we could intactly produce 2699 base long ssDNA to correctly insert 2160 bp gene cassette into the *Oxtr* locus by using this strategy (*Oxtr-T2A-iCre-ERTT2* in Table 1 and Table 2). Regarding the accuracy of ssDNA sequences, the plasmid-based nicking endonuclease method ensures the highest quality because the method is free from the enzymatic mistakes and nucleases’ side reactions [9]. However, its first step to determine appropriate nicking enzyme for the cloning of GOI into the vector and its last step with the gel separation/extraction might be challenging for beginners. The phospho-PCR method thus provides a novel practical choice to easily prepare long ssDNA donors with enough accuracy and yield for the gene cassette knock-in.

Generating long ssDNA donors at will does obviate the construction of targeting vectors with relatively long homology arms (about 2 kb at both sides). According to the previous report, 55–105 base homology arms at both sides are enough for long ssDNA donors to effectively produce knock-in mice [12]. We started to include the homology arms from approximately 300 bases long because the manufacturer’s instruction indicated the possible unintended pruning from both sides due to the exonucleases’ side reactions [24]. Nevertheless, the resulting long ssDNAs’ length was almost intact (Figure 3B), and 230–340 base homologies were enough for knock-in mouse generations (Table 2). Even when we reduced the homology arm length to 100–150 bases, the long ssDNA donors via the phospho-PCR method could work sufficiently as knock-in templates (*Tbr2-T2A-d2EGFP* in Table 2 and *Pax6-mCitrine* in Figure 4A). We would therefore recommend designing the homology arm length depending on the availability of the adequate primers for the high-fidelity PCR enzyme to yield clear single products (the details are described in Supplementary Information 1).

Here, we introduced two choices, the employment of commercially available kit or the manual production. The advantage for the commercial kit is in its stable productivity with no need of the calibration. This is probably because some sort of enhancers for exonuclease reactions might be contained in the Strandase Mix. On the other hand, the manual combination of Lambda exonuclease and Exonuclease III, which we proposed in this study, can largely reduce the cost, and the resulting ssDNA have enough qualities for knock-in mouse generation (*Pcdhb19-T2A-EGFP* in Table 1 and Table 2). However, we recommend rigorously adjusting the Exonuclease III reaction time depending on the length of ssDNA because the manually produced ssDNA tends to be slightly shorter than that produced by the kit (Figure 3B).

We also confirmed that the phospho-PCR-derived long ssDNA donors could be utilized for electroporation (Figure 4 and Figure S2). The knock-in efficiencies from these two experiments were 10.0–14.2%, which were within our range of microinjection-mediated knock-in experiments (5.9–40.0% from 12 experiments in Table 2). Although we need more practical examples to compare the effectiveness of electroporation with those of microinjection, the electroporation method might provide a much easier choice for researchers.

Importantly, we unexpectedly observed that long ssDNA donors occasionally induced incomplete insertions. For example, in Figure 4C and Figure S2B, some embryos other than those with the accurate knock-in allele yielded the shorter or longer PCR products. The frequencies of the incompletely edited alleles with long ssDNA donors tended to be higher than those with targeting vectors. The potential causes for these incomplete insertions might depend on the differences in DNA repair pathways between HR and SST-R. Hence, we recommend carefully checking the knock-in allele by the full sequencing outside from the homology arms.

Although the phospho-PCR method has left some points to be improved, it is still enough for quick *in vivo* genome editing to generate Cre/Flp recombinase, ligand-dependent inducible Cre, or fluorescent reporter expressing lines because we could obtain at least one knock-in animal from one experiment as a satisfactory rate for practical use. Incidentally, we realized no evidence of any long ssDNAs potentially inhibiting CRISPR-Cas9 activity or decreasing the number of pups born after microinjection.

Generally, Cre/Flp recombinase driver lines, exemplified by the *Dcx-T2A-iCre* line generated in Figure 2, can be employed for the spatio-temporal gene manipulation system [32,33], which we have previously developed, in combination with the recombinase-dependent viral vectors. These optogenetic strategies can contribute to systematic analysis of dynamic gene regulation changes in cells.

Our simple and speedy protocols proposed here could therefore be the first choice to easily produce error-free long ssDNA donors with a cost-effective manner in the lab, and the resulting functional gene cassette knock-in lines should accelerate biological research.

## Figures and Tables

**Table 1:** Summary of long ssDNA recovery rates in the phospho-PCR method.

Functional Gene Cassette	Gene Locus	Knock-In Cassette	Template dsDNA Preparation Method	Long ssDNA Length (bases)	Input dsDNA (PCR Products) (µg)	Output Long ssDNA (µg)	Recovery Rate ^1^ (Output ssDNA/Input dsDNA) (%)
Recombinases	*Dcx*	*T2A-iCre*	Artificially synthesized gene	1713	10.4	2.3	22.1
	*Dcx*	*T2A-FLPo*	In-fusion cloning	1959	20.6	3.6	17.5
	*Tubb3*	*T2A-iCre*	In-fusion cloning	1661	15.4	2.6	16.9
	*Tubb3*	*T2A-FLPo*	Artificially synthesized gene	1907	10.0	2.0	20.0
	*Tbr2*	*T2A-iCre*	In-fusion cloning	1735	15.0	2.7	18.0
	*Oxtr*	*T2A-iCre*	In-fusion cloning	1697	10.0	2.2	22.0
	*Oxtr*	*T2A-iCre-ERT2*	In-fusion cloning	**2699 ^2^**	10.8	2.6	24.0
Fluorescent reporters	*Nr4a2*	*IRES-DsRed*	Artificially synthesized gene	1807	11.7	2.4	20.5
	*Tbr2*	*T2A-d2EGFP*	Artificially synthesized gene	1123	9.0	1.8	20.0
	*Cdh11*	*EGFP*	In-fusion cloning	1380	12.6	1.9	15.0
	*Pcdhb19*	*T2A-tdTomato*	In-fusion cloning	2173	13.8	3.0	21.7
	*Pcdhb19*	*T2A-EGFP*	In-fusion cloning	1459	10.9	1.9	18.0

^1^ Long ssDNA recovery rates were calculated by dividing output long ssDNA (µg) by input dsDNA (µg). Note that long ssDNA recovery rates in the phospho-PCR method (approximately 20%) were higher than in the previously reported other methods, such as the nicking endonuclease method. ^2^ The longest ssDNA donor we generated in this study was 2699 bases long.

**Table 2:** Summary of knock-in mouse generations by using the phospho-PCR-mediated long ssDNA donors.

Knock-In Allele ^1^		Knock-In Cassette Length (bases)	Homology Arms’ Length (bases)	Injected ssDNA Conc. (ng/uL)	Injected, Survived, and Transferred Eggs	Newborn Pups (NB)	Sequence-Verified Precise knock-In Pups (KI)	Efficiency ^2^ (KI/NB) (%)
Recombinases	*Dcx-T2A-iCre*	1116	293, 304	19.2	155	14	4	28.5
	*Dcx-T2A-FLPo*	1362	293, 304	24.0	62	10	2	20.0
	*Tubb3-T2A-iCre*	1116	307, 238	17.6	68	5	2	40.0
	*Tubb3-T2A-FLPo*	1362	307, 238	17.4	136	28	2	7.1
	*Tbr2-T2A-iCre*	1125	297, 313	24.8	60	13	3	23.0
	*Oxtr-T2A-iCre*	1158	245, 294	18.5	74	11	1	9.0
	*Oxtr-T2A-iCre-ERT2*	**2160 ^3^**	245, 294	20.0	36	15	1	6.6
Fluorescent reporters	*Nr4a2-IRES-DsRed*	1277	257, 273	25.1	49	5	1	20.0
	*Tbr2-T2A-d2EGFP*	1125	**102, 106 ^4^**	15.7	100	17	1	5.9
	*Cdh11-EGFP*	714	253, 413	27.0	81	17	1	5.9
	*Pcdhb19-T2A-tdTomato*	1497	347, 329	27.4	92	24	2	8.3
	*Pcdhb19-T2A-EGFP*	786	344, 329	23.7	46	12	1	8.3

^1^ All the knock-in alleles listed above were confirmed in live-born animals, and the alleles were successfully transmitted to the next generations. ^2^ Knock-in efficiencies were calculated by dividing the number of sequence-verified knock-in pups by the number of newborn pups. ^3^ The longest knock-in length we achieved in this study was 2160 bases long. ^4^ The shortest homology arms’ length we confirmed to be sufficient in this study was 102 and 106 bases long.

## Data Availability

The data presented in this study are available in this article and Supplementary Materials.

## Supplementary Materials

The following are available online at https://www.mdpi.com/article/10.3390/cells10051076/s1. Figure S1: Three strategies to prepare long ssDNA donors in the lab. Figure S2: Long ssDNA donors prepared by the phospho-PCR method could be used for electroporation-mediated Cre recombinase knock-in. Table S1: CRISPR RNAs used in this study. Table S2: PCR primers used in this study. Supplementary Information 1. Designing dsDNA templates for the phospho-PCR methods. Supplementary Information 2. Optimization of PCR conditions to amplify pure single product as exonuclease substrates. Supplementary Information 3. Manual ssDNA production by utilizing Lambda exonuclease and Exonuclease III.

## Abbreviations

CRISPR/Cas9	clustered regularly interspaced short palindrome repeats/CRISPR-associated protein-9 nuclease
ssDNA	single-stranded DNA
FACS	fluorescence-activated cell sorting
crRNA	CRISPR RNA
tracrRNA	trans-activating crRNA
DSB	double-strand break
HDR	homology-directed repair
HR	homologous recombination
dsDNA	double-stranded DNA
SSTR	single strand template repair
ivTRT	in vitro transcription and reverse transcription
SNP	single nucleotide polymorphism
KI	knock-in
Dcx	doublecortin
GOI	gene cassette of interest
iCre	codon-improved version of Cre recombinase
FLPo	codon-optimized version of enhanced flippase
ERT2	mutated human estrogen receptor ligand binding domain
