# Supplementary material for: An Optimized Preparation Method for Long ssDNA Donors to Facilitate Quick Knock-In Mouse Generation
Source: Cells. 2021 Apr 30;10(5):1076. doi: 10.3390/cells10051076 (PMC8147208; doi:10.3390/cells10051076)
Supplement: Supplementary file 1 [file cells-10-01076-s001.zip › cells-1180729-supplementary.pdf]

cells

Article

An Optimized Preparation Method for Long ssDNA Donors to Facilitate Quick Knock-In Mouse Generation

Yukiko U. Inoue [1,*], Yuki Morimoto [1], Mayumi Yamada [2], Ryosuke Kaneko [3], Kazumi Shimaoka [1], Shinji Oki [4], Mayuko Hotta [1], Junko Asami [1], Eriko Koike [1], Kei Hori [1], Mikio Hoshino [1], Itaru Imayoshi [2,5] and Takayoshi Inoue [1,*]

[1] Department of Biochemistry and Cellular Biology, National Institute of Neuroscience, National Center of Neurology and Psychiatry, Ogawahigashi, Kodaira, Tokyo 187-8502, Japan; 99a117@gmail.com (Y.M.); kshimaoka@ncnp.go.jp (K.S.); mhotta@ncnp.go.jp (M.H.); asami@ncnp.go.jp (J.A.); koikeike@ncnp.go.jp (E.K.); khori@ncnp.go.jp (K.H.); hoshino@ncnp.go.jp (M.H.)
[2] Research Center for Dynamic Living Systems, Graduate School of Biostudies, Kyoto University, Kyoto 606-8501, Japan; yamada.mayumi.4a@kyoto-u.ac.jp (M.Y.); imayoshi.itaru.2n@kyoto-u.ac.jp (I.I.)
[3] KOKORO-Biology Group, Laboratories for Integrated Biology, Graduate School of Frontier Biosciences, Osaka University, Suita, Osaka 565-0871, Japan; rkaneko@fbs.osaka-u.ac.jp (R.K.)
[4] Department of Immunology, National Institute of Neuroscience, National Center of Neurology and Psychiatry, Ogawahigashi, Kodaira, Tokyo 187-8502, Japan; soki@ncnp.go.jp (S.O.)
[5] Department of Deconstruction of Stem Cells, Institute for Frontier Life and Medical Sciences, Kyoto University, Kyoto 606-8507, Japan
* Correspondence: yinn3@ncnp.go.jp (Y.U.I.); tinoue@ncnp.go.jp (T.I.)

Citation: Inoue, Y.U.; Morimoto, Y.; Yamada, M.; Kaneko, R.; Shimaoka, K.; Oki, S.; Hotta, M.; Asami, J.; Koike, E.; Hori, K.; et al. An Optimized Preparation Method for Long ssDNA Donors to Facilitate Quick Knock-In Mouse Generation. *Cells* **2021**, *10*, 1076. https://doi.org/10.3390/cells10051076

Academic Editor: Alexander E. Kalyuzhny

Received: 28 March 2021
Accepted: 28 April 2021
Published: 30 April 2021

Publisher's Note: MDPI stays neutral with regard to jurisdictional claims in published maps and institutional affiliations.

Copyright: © 2021 by the authors. Licensee MDPI, Basel, Switzerland. This article is an open access article distributed under the terms and conditions of the Creative Commons Attribution (CC BY) license (http://creativecommons.org/licenses/by/4.0/).

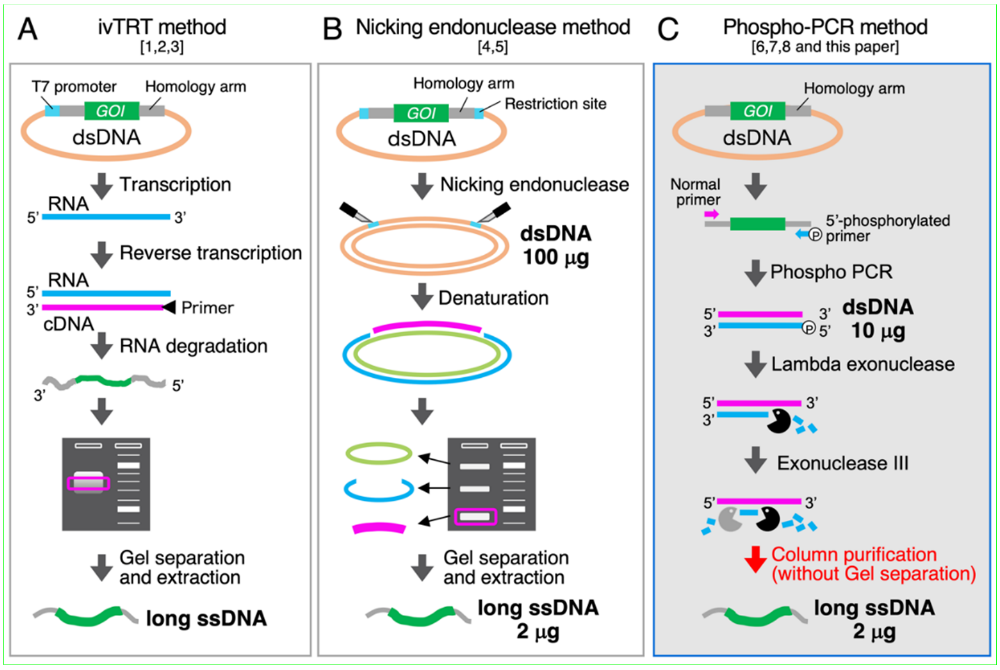

Figure S1. Three strategies to prepare long ssDNA donors in the lab. (**A**) ivTRT represents *in vitro* transcription and reverse transcription [1-3]. The template dsDNA is first transcribed into RNA by T7 RNA polymerase, and then reverse transcription is performed to yield RNA/DNA hybrids. By degrading the RNA strand by RNase treatment, long ssDNA donors can be prepared. For pure ssDNAs to be obtained, gel separation and extraction steps are needed. (**B**) The nicking endonuclease method is based on the double nicking of plasmid DNA [4,5]. The objective fragment is first cloned into a plasmid vector using nicking endonuclease sites, and then the plasmid is digested by the enzymes. The nicked plasmid is denatured and subjected to agarose gel electrophoresis. The band corresponding to long ssDNA is excised and extracted. For 2 µg of long ssDNA to be obtained, 100 µg of plasmid needs to be digested. (**C**) In the phospho-PCR method [6-8], the substrate dsDNAs for exonuclease reactions are amplified by a pair of normal primer and 5′-phosphorylated primer. First, Lambda exonuclease selectively digests the phosphorylated chains of the duplex to yield ssDNAs. Subsequently added Exonuclease III helps the completion of ssDNA production. The resulting

ssDNA can be easily purified by using spin-column without laborious gel separation steps. For 2 µg of long ssDNA to be obtained, only 10 µg of dsDNA needs to be digested. Abbreviations: GOI, gene cassette of interest; dsDNA, double-stranded DNA; ssDNA, single-stranded DNA.

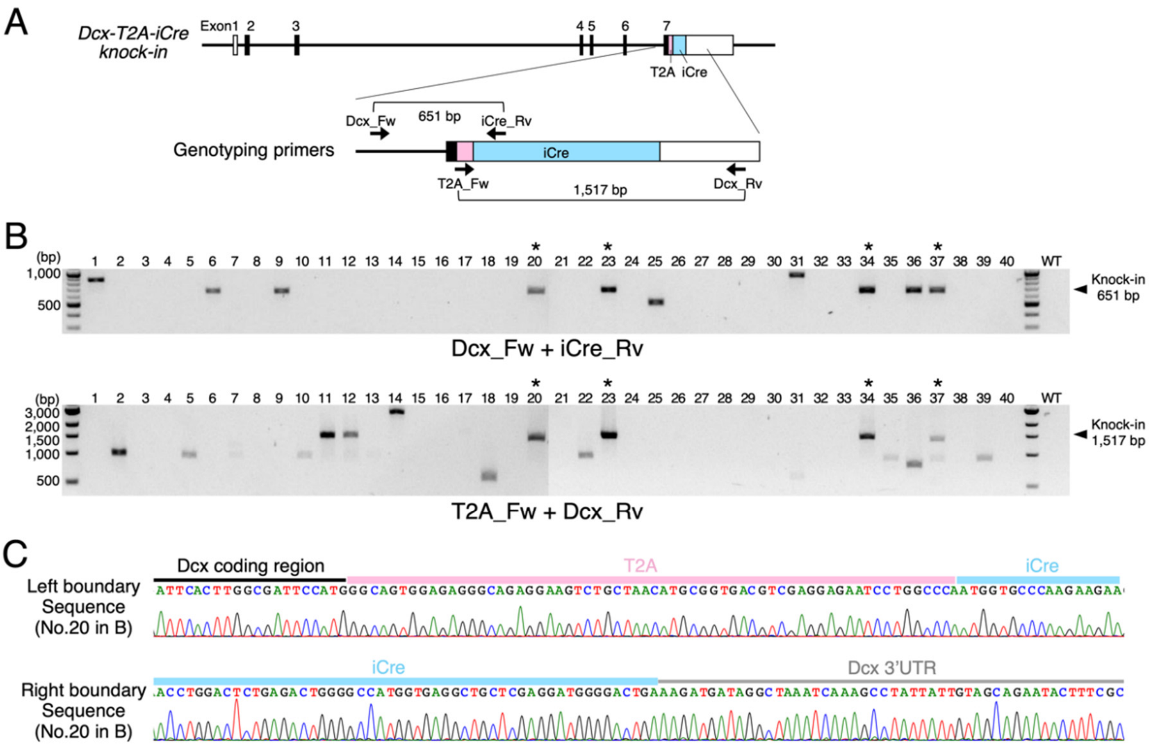

Figure S2. Long ssDNA donors prepared by the phospho-PCR method could be used for electroporation-mediated Cre recombinase knock-in. (**A**) *Dcx-T2A-iCre* knock-in allele (same as Figure 2A) and the genotyping primers are depicted. Dcx_Fw and Dcx_Rv were designed outside from the donor DNA's homology arms to exclude the detection of unintended random integrations. (**B**) PCR screening for blastocysts derived from the electroporation using long ssDNA donor. Asterisks indicate the sequence-verified knock-in embryos with correct T2A-iCre insertion (No.20, 23, 34, and 37). (**C**) Boundary sequences between the *Dcx* gene and T2A-iCre cassette analyzed by using the genome DNA from No.20 embryo in (B) are aligned.

Table S1. CRISPR RNAs used in this study.

Locus	Name	Supplier	Sequence (5' to 3')
All the loci	tracrRNA	FASMAC	AAACAGCAUAGCAAGUUAAAAU-AAGGCUAGUCCGUUAUCAACUUGAAAAAGUG-GCACCGAGUCGGUGCU
	tracrRNA	IDT[1]	AGCAUAGCAAGUUAAAAU-AAGGCUAGUCCGUUAUCAACUUGAAAAAGUG-GCACCGAGUCGGUGCUUU (with modifications to block endonucleases'/exonucleases' attacks)
Dcx	Dcx-crRNA	FASMAC	UUAGCCUAUCAUCUUUCACAguuuuaga-gcuaugcuguuuug
Dcx	Dcx-crRNA	IDT[1]	AltR1[2]/UUAGCCUAUCAUCUUUCACAguuuuaga-gcuaugcu/AltR2[2]
Tubb3	Tubb3-crRNA	FASMAC	AGCUGCGAGCAACUUCACUUguuuuaga-gcuaugcuguuuug
Tbr2	Tbr2-crRNA	FASMAC	AAAGGUUAAAAUAAUGCUCUguuuuaga-gcuaugcuguuuug
Oxtr	Oxtr-crRNA	FASMAC	CCAUCCUCGGCAUGAGCCAUguuuuaga-gcuaugcuguuuug

Nr4a2	Nr4a2-crRNA	FASMAC	CUUGGGAGAAGGUCUUAGAAguuuuaga-gcuaugcuguuuug
Cdh11	Cdh11-crRNA	FASMAC	GUCAUCAUCAAAAGUGUCUUguuuuaga-gcuaugcuguuuug
Pcdhb19	Pcdhb19-crRNA	FASMAC	CAAUAGUUUUGGGUUAUUUAguuuuaga-gcuaugcuguuuug
Pax6	Pax6-crRNA	IDT[1]	AltR1[2]/UCGAUUACAGUAAAGAGAGAguuuuaga-gcuaugcu/AltR2[2]

[1] IDT: integrated DNA technologies; [2]AltR1, AltR2: modifications to block exonucleases' attacks.

Table S2. PCR primers used in this study.

Figure	Knock-In Allele	Primer Name	Sequence (5' to 3')
Figure 2A, 2C Figure S2A, S2B (genotyping)	*Dcx-T2A-iCre*	Dcx_Fw	GGCCACTAGTATTCCCAGGAAC
		iCre_Rv	GTCCCTGAACATGTCCATCAGGTTC
		T2A-_Fw	GAAGTCTGCTAACATGCGGTGAC
		Dcx_Rv	CAGCCTTCCAGAGAAGGAAGAC
Figure 2B (phospho-PCR)	*Dcx-T2A-iCre*	5P-Dcx_Fw2	5'Phos/ TCACACAAATAA-GAACATGCCAG
		Dcx_Rv2	TAGTTTCTGAACAAATTGAGTGGG
Figure 4A, 4C (genotyping)	Pax6-mCitrine	Pax6_Fw	CGTAGAATCTACCTATGCGGATGGTG
		mCitrine_C_Rv	AACTCCAGCAGGACCATGTGATC
		mCitrine_N_Fw	TAAACGGCCACAAGTTCAGCGTGTC
		Pax6_Rv	GCAAAGGTCCTTGGTTCTAGTCCATTC

Supplementary Information

1. Designing dsDNA Templates for the Phospho-PCR Methods

According to the previous report, 55–105 base long arms homologous to the genome sequence flanking the targeted site of integration are enough for long ssDNA donors to effectively produce knock-in mice. In the present study, 102–413 base homology arms were also shown to be sufficient (Table 2). To determine the length of homology arms, we need to specifically consider availabilities of adequate primers for the high-fidelity PCR enzyme, PrimeSTAR Max DNA Polymerase (Takara Bio), instead of sticking with the homology arms' "length". To obtain clear single dsDNA substrates for the exonuclease reactions by PCR, we recommend designing primers that are 24–27 bases long with relatively higher melting temperature (Tm) (60–62 °C). The manufacturer's instructions could be informative (https://www.takarabio.com/documents/User%20Manual/R045A/R045A_e.v1510Da.pdf) (accessed on 30 April 2021).

Next, the initial dsDNA templates for the phospho-PCR can be designed by arranging the homology arms determined above at both sides of gene cassette of interests (GOI). In ordering the artificial synthesis of the designed dsDNA, we recommend choosing the sequence-verified cloned gene because the cheaper gene blocks without cloning services sometimes contain the incorrect bases. Similarly, in constructing the dsDNA templates by yourself employing In-Fusion cloning, all the bases are needed to be checked by full sequencing.

2. Optimization of PCR Conditions to Amplify Pure Single Product as Exonuclease Substrates

There are two choices for primer combinations: 5'-phospholylated forward primer (5P-F) and normal reverse primer (R) to yield antisense ssDNA or normal forward primer (F) and 5'-phospholylated reverse primer (5P-R) to yield sense ssDNA. In many cases, little difference between the two has been noticed in terms of PCR product yields, long ssDNA yields, and knock-in efficiencies obtained. In case of any problems with one primer combination, it is possible to switch it to another.

As shown in Figure S1-C, 10 μg of dsDNA should be digested to obtain 2 μg of long ssDNA. We usually obtain 10 μg of dsDNA can be amplified from 200 μL of PCR mixture. Before scaling up the reaction mixture to 200 μL, we recommend performing the PCR optimization by using 10 μL mixture for each condition described below.

Starting the primers' concentrations at 0.2 μM and the annealing temperature at 60°C are recommended by using the adequate primer sequences described in Supplementary Information 1. For the template concentration to be rigorously optimized, dilution series of [500 pg/μL, 5 pg/μL, and 2.5 pg/μL] for the final concentrations of [25 pg/μL, 0.25 pg/μL, and 0.125 pg/μL] are usually prepared. In many cases, final concentrations of 25 pg/μL or 0.25 pg/μL were noticed to be suitable to amplify pure single products. In cases that there are still observed non-specific amplifications, the annealing temperature should be raised to 62 °C or 63 °C.

In scaling up the PCR mixture to 200 μL after determining the best condition, aliquoting 200 μL into eight tubes (25 μL each) is recommended to obtain enough PCR products by ensuring the temperature control. This extra effort is also important in preparing concentrated long ssDNA for electroporation by scaling up the PCR mixture.

For the electroporation-mediated knock-in mouse generation, the volume of initial PCR mixture could be scaled up more than five times, and the following exonuclease reactions could be performed in five tubes in parallel. To concentrate the long ssDNAs, they were collected into one tube and precipitated by using isopropanol. After the pellet is washed with 75% ethanol, it can be dissolved in a small amount of OptiMEM I (approximately 5 μL) by heating the tube at 55 °C. Gentle yet more than 100 times' pipetting might be needed to completely dissolve the pellet. To check the concentration and the integrity, we can dilute a tiny amount of the concentrated ssDNA for the NanoDrop (Thermo Fisher Scientific, Waltham, MA, U.S.A.) measurement and the agarose gel electrophoresis.

3. Manual ssDNA Production by Utilizing Lambda Exonuclease and Exonuclease III

The starting substrates can be amplified by the phospho-PCR as described in Supplementary Information 2. For microinjection, 10 μg of dsDNA substrates from 200 μL of PCR mixture is usually prepared to obtain 2 μg of long ssDNA. The PCR products were purified by using NucleoSpin column (Takara Bio). A small portion of the mixture was used to measure the concentration on NanoDrop, and the amounts of products were calculated. First, 10 units of Lambda exonuclease (New England BioLab M0262) were added to 10 μg of dsDNA substrates to digest 5'-phospholyrated chains of dsDNAs. After 30 min incubation at 37 °C, the reaction mixture was heated at 75 °C, 10 min, to inactivate the enzyme. Next, 90 units of Exonuclease III (Takara Bio 2170A) were added to the reaction mixture to complete the ssDNA production. The incubation time should be rigorously adjusted depending on the starting dsDNA's length. As a standard, the reaction mixture is usually incubated for 1 min per 300 bases at 37 °C. After inactivating the enzyme at 65 °C, 5 min, we checked a small portion of the reaction mixture on the agarose gel electrophoresis to confirm the ssDNA purity. Then, the remaining mixture was purified by NucleoSpin column (Takara Bio) to obtain ssDNAs. For microinjections, ssDNAs were eluted by 12 μL of 0.1× TE buffer (described in the Materials and Methods section). To increase the ssDNA recovery, we pre-heated 0.1× TE buffer (70 °C) before use, and the column was incubated for 5 min before the spin down. The ssDNA concentration was determined by analyzing 1 μL of the eluted solution on NanoDrop. The concentration could be 150–200 ng/μL. We preserved 5 μL aliquots at –80 °C until use.

Supplementary References

1. Miura, H.; Gurumurthy, C.B.; Sato, T.; Sato, M.; Ohtsuka, M. CRISPR/Cas9-based generation of knockdown mice by intronic insertion of artificial microRNA using longer single-stranded DNA. *Sci. Rep.* **2015**, *5*, 1–11, doi:10.1038/srep12799.
2. Yoshimi, K.; Kunihiro, Y.; Kaneko, T.; Nagahora, H.; Voigt, B.; Mashimo, T. SsODN-mediated knock-in with CRISPR-Cas for large genomic regions in zygotes. *Nat. Commun.* **2016**, *7*, doi:10.1038/ncomms10431.

3. Quadros, R.M.; Miura, H.; Harms, D.W.; Akatsuka, H.; Sato, T.; Aida, T.; Redder, R.; Richardson, G.P.; Inagaki, Y.; Sakai, D.; et al. Easi-CRISPR: A robust method for one-step generation of mice carrying conditional and insertion alleles using long ssDNA donors and CRISPR ribonucleoproteins. *Genome Biol.* **2017**, *18*, 92, doi:10.1186/s13059-017-1220-4.
4. Miyasaka, Y.; Uno, Y.; Yoshimi, K.; Kunihiro, Y.; Yoshimura, T.; Tanaka, T.; Ishikubo, H.; Hiraoka, Y.; Takemoto, N.; Tanaka, T.; et al. CLICK: One-step generation of conditional knockout mice. *BMC Genom.* **2018**, *19*, 318, doi:10.1186/s12864-018-4713-y.
5. Miura, H.; Quadros, R.M.; Gurumurthy, C.B.; Ohtsuka, M. Easi-CRISPR for creating knock-in and conditional knockout mouse models using long ssDNA donors. *Nat. Protoc.* **2018**, *13*, 195–215, doi:10.1038/nprot.2017.153.
6. Aida, T. Genome Editing in Mice. In Proceedings of the Japanese Society for Genome Editing, Satellite Symposium "Practical Genome Editing", Osaka, Japan, 1 July 2017.
7. Takara Bio. Highly Specific Gene Knockins of Long Sequences using CRISPR/Cas9 and a Single Stranded DNA Donor Template. 2017. Available online: https://catalog.takara-bio.co.jp/PDFS/Highly-specific-gene-knockins-of-long-sequences-using-CRISPR_Cas9-and-a-single-stranded-DNA-donor-template.pdf (accessed on 25 March 2021).
8. Nakade, S.; Aida, T. Single-stranded DNA donors for CRISPR knock-in. In *Standard Protocols on Genome Editing*; Yamamoto, T., Sakuma, T., Eds.; YODOSHA: Tokyo, Japan, 2019; pp. 41–49. ISBN 978-4-7581-2244-3.
